# Treatment Modalities and Recurrence Outcomes in Odontogenic Keratocysts: A 24-Year Retrospective Analysis

**DOI:** 10.3390/healthcare14131834

**Published:** 2026-06-24

**Authors:** Nur Efşan Aydın, Özgür Dağal, Nur Mollaoğlu

**Affiliations:** Department of Oral and Maxillofacial Surgery, Faculty of Dentistry, Gazi University, 06000 Ankara, Turkey; ozgurdagal@gazi.edu.tr (Ö.D.); nurmolla@gazi.edu.tr (N.M.)

**Keywords:** odontogenic keratocyst, recurrence, follow-up, treatment outcomes

## Abstract

**Highlights:**

**What are the main findings?**

- Enucleation showed the highest recurrence rate among treatment modalities for odontogenic keratocysts.
- Marsupialization was associated with a lower recurrence rate in this cohort.

**What are the implications of the main findings?**

- Conservative treatment approaches may be effective in selected odontogenic keratocyst cases.
- Long-term clinical and radiological follow-up remains essential because of the potential for delayed recurrence.

**Abstract:**

**Background:** Odontogenic keratocysts are developmental cysts of the jaws that often remain asymptomatic until they reach considerable size and are most frequently located in the mandibular angle and ramus regions. Due to their high recurrence potential, the optimal treatment approach remains controversial. The aim of this study was to evaluate treatment modalities associated with lower recurrence rates in odontogenic keratocysts. **Material and Methods:** Patients diagnosed with odontogenic keratocyst between 2000 and 2024 at the Department of Oral and Maxillofacial Surgery, Gazi University, were retrospectively evaluated. Associations between gender, age, lesion localization, histological subtype, treatment modality, and recurrence were analyzed. Statistical analyses were performed using SPSS for Windows (version 27). **Results:** A total of 291 cases were included, with an overall recurrence rate of 16.2%. The highest recurrence rate was observed in patients treated with enucleation (19.2%), whereas a lower recurrence rate was found in cases treated with marsupialization (5%). No recurrence was observed in patients who underwent resection. A statistically significant association was found between treatment modality and recurrence (*p* = 0.014). **Conclusions:** Treatment selection for odontogenic keratocysts should be carefully planned. In the present study, marsupialization was associated with a lower recurrence rate than enucleation in selected cases. However, because of the retrospective design and non-randomized treatment allocation, these findings should be interpreted with caution and should not be considered evidence of a causal relationship. Long-term clinical and radiological follow-up remains essential because of the potential for late recurrence.

## 1. Introduction

Odontogenic keratocyst (OKC) is a developmental odontogenic cyst originating from remnants of the dental lamina and is currently classified as a cyst according to the 2022 World Health Organization (WHO) Classification of Head and Neck Tumours [1,2,3]. Histologically, OKC is characterized by a keratinized epithelial lining and has traditionally been classified into orthokeratinized and parakeratinized subtypes, the latter being associated with more aggressive clinical behavior and a higher recurrence potential [4,5].

OKCs account for approximately 3–11% of all jaw cysts and occur more frequently in males. They are most commonly located in the posterior mandible, particularly in the angle and ramus regions, although they may occur throughout the jaws [5,6]. Radiographically, they typically present as well-defined unilocular or multilocular radiolucent lesions and may mimic other odontogenic cysts, particularly dentigerous cysts [7].

Despite their benign nature, OKCs are characterized by a relatively high recurrence rate, making treatment planning and long-term follow-up challenging. Numerous treatment modalities have been proposed, ranging from conservative approaches such as enucleation and marsupialization to more aggressive procedures including peripheral ostectomy, chemical cauterization, cryotherapy, and resection [8]. More recently, adjunctive therapies such as 5-fluorouracil have also been investigated as potential strategies to reduce recurrence [9]. However, the optimal management of OKCs remains controversial, and treatment decisions are often influenced by factors such as lesion size, anatomical location, histological subtype, and previous treatment history.

Therefore, the aim of the present study was to retrospectively evaluate patients diagnosed with odontogenic keratocyst between 2000 and 2024 at the Department of Oral and Maxillofacial Surgery, Gazi University. The study assessed demographic and clinical characteristics, treatment modalities, recurrence outcomes, and factors associated with recurrence. In addition, long-term clinical outcomes from a relatively large single-center cohort were analyzed and recurrence patterns among different treatment approaches were compared.

## 2. Materials and Methods

In this study, all cases diagnosed with odontogenic keratocyst (OKC) between January 2000 and January 2024 were retrospectively reviewed. The diagnoses of all included cases were based on histopathological reports. Histopathological evaluations issued by the Department of Pathology were re-assessed according to the diagnostic criteria defined in the World Health Organization (WHO) 2022 Classification of Head and Neck Tumours. Ethical approval for this retrospective study was obtained from the Ethics Commission of the Rectorate of Gazi University (Approval Number: 2024–1186), and all procedures were performed in accordance with the Declaration of Helsinki.

The follow-up protocol consisted of clinical and radiographic examinations every three months during the first postoperative year and annually thereafter, with a minimum intended follow-up period of five years. Follow-up duration was calculated as the interval between the date of primary surgical treatment and the date of the last documented clinical or radiographic evaluation. For patients with recurrence, the time to recurrence was recorded as the interval between the date of primary treatment and the date on which recurrence was first detected clinically and/or radiographically.

For localization analysis, the jaws were divided into two anatomical regions: maxilla and mandible. The anterior region was defined as the canine-to-canine segment, while the posterior region included the premolar, molar, ramus, and tuberosity areas. Lesion dimensions were obtained from preoperative radiographic records, and the maximum lesion width and length were recorded in millimeters.

The surgical treatment modalities were categorized based on operative notes and medical records and defined according to standard descriptions in the literature. Marsupialization was defined as the creation of a surgical window in the cystic wall, followed by suturing of the cyst lining to the oral mucosa to allow continuous decompression of the lesion. This approach was primarily selected for large lesions, lesions in close proximity to vital anatomical structures, or cases in which a more conservative initial approach was considered appropriate. The decision to proceed to secondary definitive surgery after marsupialization was based on clinical and radiographic evidence of lesion shrinkage, new bone formation, and reduced surgical risk to adjacent anatomical structures.

Enucleation was defined as the complete removal of the cystic lesion together with the cyst lining, without intentional removal of the surrounding bone, and was generally preferred for smaller lesions with well-defined borders. Resection was defined as the removal of the lesion along with a margin of surrounding bone and was performed as a marginal or segmental resection depending on lesion extent and anatomical involvement, particularly in extensive, recurrent, or aggressive cases. Information regarding the use of adjunctive treatments, such as peripheral ostectomy or chemical cauterization, was not consistently available in the archival records and therefore could not be included in the analysis.

In our institution, marsupialization is generally used as an initial decompression procedure and is commonly followed by secondary enucleation after sufficient lesion shrinkage. Due to the retrospective nature of the study, details regarding subsequent procedures were not consistently available for all patients and therefore treatment groups were classified according to the initial treatment approach.

Recurrence was defined as the detection of a new cystic lesion at the site of previous treatment during follow-up after the primary surgical procedure, based on clinical and/or radiographic findings. The diagnosis of recurrence was established through clinical examination and panoramic radiographic evaluation. Histopathological confirmation was performed when available. Cases without recurrence during the follow-up period were considered recurrence-free up to the date of the last follow-up.

Each patient was included only once in the study cohort. Treatment groups were defined according to the initial treatment modality applied at first presentation. Recurrent lesions requiring additional surgical interventions during follow-up were not entered into the database as separate cases.

The variables included in the analyses were age, sex, lesion localization, histopathological subtype, lesion dimensions, treatment modality, follow-up duration, time to recurrence, and recurrence status.

Statistical analyses were performed using the Statistical Package for the Social Sciences (SPSS) for Windows, version 27 (SPSS Inc., Chicago, IL, USA), and R software (R Foundation for Statistical Computing, Vienna, Austria; Version 4.3.1). Descriptive statistics were presented as frequencies, percentages, means, standard deviations, medians, and interquartile ranges (IQRs), as appropriate. Associations between categorical variables were evaluated using the Chi-square test. When the assumptions of the Chi-square test were not met, Fisher’s Exact Test was used for 2 × 2 contingency tables, and the Fisher–Freeman–Halton Exact Test was applied for larger R × C tables.

To identify factors associated with recurrence, univariate logistic regression analyses were initially performed. Due to the low number of events in certain categories and the presence of complete separation, multivariable analyses were conducted using Firth’s penalized logistic regression method. Results were reported as odds ratios (ORs), 95% confidence intervals (CIs), and *p*-values.

To account for differences in follow-up duration and to evaluate the time to recurrence, survival analyses were performed. Recurrence was defined as the event of interest, whereas cases without recurrence during the follow-up period were treated as censored observations. Recurrence-free survival probabilities were estimated using the Kaplan–Meier method, and mean and median recurrence-free survival times were reported together with their 95% confidence intervals.

To determine factors associated with recurrence-free survival, univariate and multivariable Cox proportional hazards regression analyses were performed. The proportional hazards assumption of the Cox model was assessed using Schoenfeld residuals. A two-sided *p*-value < 0.05 was considered statistically significant for all analyses.

## 3. Results

A total of 291 patients diagnosed with odontogenic keratocyst (OKC) were included in this study. Of the patients, 64.9% were male, and the majority of lesions were located in the posterior mandible (70.8%). Enucleation was the most frequently performed treatment modality (80.4%), and the parakeratinized histopathological subtype was predominant (92.8%). The overall recurrence rate was 16.2% (Table 1).

No statistically significant association was observed between recurrence and age group or lesion localization (*p* = 0.215 and *p* = 0.698, respectively) (Table 2 and Table 3). Similarly, no significant association was identified between histopathological subtype and recurrence (*p* = 0.353) (Table 4). The relationship between pre-diagnosis and recurrence was also not statistically significant (*p* = 0.328) (Table 5).

A statistically significant association was found between treatment modality and recurrence (*p* = 0.014) (Table 6). The highest recurrence rate was observed in the enucleation group (19.2%), whereas the recurrence rate in the marsupialization group was 5%. No recurrence was detected among patients treated with resection. Among recurrence-positive cases, 95.7% had undergone enucleation and 4.3% had undergone marsupialization.

The distribution of follow-up duration, time to recurrence, and lesion dimensions according to treatment modality is presented in Table 7. The mean follow-up duration was 56.49 ± 13.18 months in the enucleation group, 59.30 ± 9.02 months in the marsupialization group, and 71.18 ± 5.89 months in the resection group. Lesion width and lesion length were greater in the marsupialization and resection groups than in the enucleation group.

To identify factors associated with recurrence, univariate and multivariable Firth logistic regression analyses were performed (Table 8). In the univariate analysis, being aged 51–60 years (OR = 0.213, 95% CI: 0.032–0.808, *p* = 0.047), treatment with marsupialization (OR = 0.221, 95% CI: 0.035–0.760, *p* = 0.043), longer follow-up duration (OR = 1.109, 95% CI: 1.066–1.160, *p* < 0.001), lesion width (OR = 0.905, 95% CI: 0.828–0.964, *p* = 0.009), and lesion length (OR = 0.925, 95% CI: 0.861–0.972, *p* = 0.010) were significantly associated with recurrence. In the multivariable analysis, only follow-up duration remained an independent predictor of recurrence (OR = 1.132, 95% CI: 1.079–1.196, *p* < 0.001).

**Figure 1:**
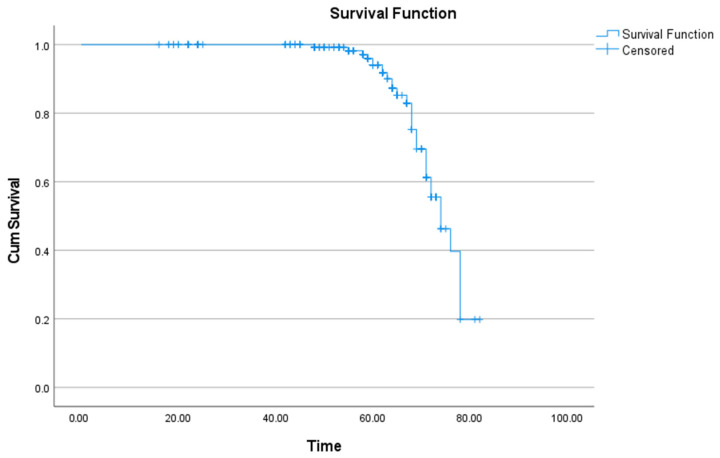
Kaplan–Meier Curve for Recurrence-Free Survival.

Kaplan–Meier survival analysis was performed to evaluate the temporal pattern of recurrence. The mean recurrence-free survival time was 73.31 months (95% CI: 71.67–74.94 months), while the median recurrence-free survival time was 74 months (95% CI: 70.18–77.82 months) (Figure 1, Table 9).

The proportional hazards assumption of the Cox regression model was assessed using Schoenfeld residuals. No violation of the proportional hazards assumption was detected for sex, age, localization, pre-diagnosis, histopathological subtype, treatment modality, lesion width, or lesion length (all *p* > 0.05). Furthermore, the global test confirmed that the proportional hazards assumption was satisfied for the model as a whole (Global χ^2^ = 11.300, df = 18, *p* = 0.880) (Table 10).

Univariate and multivariable Cox proportional hazards regression analyses were performed to identify factors associated with recurrence-free survival (Table 11). In the univariate analysis, being aged 31–40 years (HR = 2.358, 95% CI: 1.024–5.431, *p* = 0.044), longer follow-up duration (HR = 1.109, 95% CI: 1.066–1.160, *p* < 0.001), smaller lesion width (HR = 0.906, 95% CI: 0.856–0.958, *p* < 0.001), and smaller lesion length (HR = 0.930, 95% CI: 0.891–0.970, *p* < 0.001) were significantly associated with recurrence timing. In the multivariable analysis, marsupialization was associated with a reduced risk of recurrence (HR = 0.020, 95% CI: 0.001–0.680, *p* = 0.030), whereas longer follow-up duration was associated with an increased risk of recurrence (HR = 1.132, 95% CI: 1.079–1.196, *p* < 0.001). No other variables demonstrated an independent association with recurrence-free survival (all *p* > 0.05).

## 4. Discussion

Odontogenic cysts are usually detected during routine examinations and may be of inflammatory or developmental origin. Odontogenic keratocyst (OKC), which accounts for approximately 12–14% of all odontogenic cysts, has attracted interest due to its high recurrence rate and characteristic histopathological features [5,10]. OKC originates from the dental lamina and is histologically characterized by a palisaded basal cell layer and a parakeratinized stratified squamous epithelial lining [11].

Jones et al. reported that keratocysts were more frequently observed in males [12]. In contrast, Padmakumar et al. and Avelar et al. did not find a significant association between gender and OKC occurrence [13,14]. In the present study, similar to previous research, 35.1% of cases were female and 64.9% were male, with no significant association between gender and recurrence (*p* > 0.05).

Although OKCs can occur between ages 7 and 93, they are most commonly found in the 2nd–3rd and 5th–7th decades [5]. Chirapathomsakul et al. found that OKCs were most prevalent between 11–40 years [15]. Jung et al. showed that despite a broad age range (7–84), more than half of cases occurred in the 3rd–4th decades [16]. Similarly, in this study, most cases were between 31–50 years, with no significant association between age and recurrence (*p* > 0.05).

OKCs are most commonly located in the mandible, especially in the angle and ascending ramus; in the maxilla, they appear most frequently in the third molar area [7]. Jung et al. reported that 63.2% of lesions were located in the mandibular ramus and posterior regions [16]. Urs et al. found that 76% of lesions were mandibular [17]. In this study, 80.4% of lesions were mandibular and no significant association was found between localization and recurrence (*p* > 0.05).

Defining the histological features is essential for OKC diagnosis; however, infection may obscure classical findings, causing similarities to dentigerous cysts or unicystic ameloblastomas. Kitisubkanchana et al. reported that a unilocular radiolucent lesion without root resorption is more suggestive of OKC than ameloblastoma [18]. Cai et al. demonstrated that digital pathology-based AI models offer promising diagnostic performance for OKCs [19].

The orthokeratinized subtype accounts for ~10% of cases, with prevalence varying slightly by population [5]. Urs et al. reported that 95% of their cases exhibited parakeratinization, which was associated with higher recurrence [17]. In the present study, 92.8% were parakeratinized and 7.2% were orthokeratinized. Nevoid basal cell carcinoma syndrome (NBCCS) is characterized by multiple basal cell carcinomas, multiple jaw cysts, skeletal anomalies, and ectopic calcifications. In the present study, only one patient was associated with NBCCS. González-Alva et al. reported NBCCS association in only 6% of cases [20]. Because only a single NBCCS-associated case was identified in the present cohort, no meaningful statistical comparison could be performed. Nevertheless, the presence of syndromic cases should be considered when interpreting recurrence patterns, as OKCs associated with NBCCS have been reported to demonstrate distinct biological behavior and a higher tendency for multiplicity and recurrence.

The optimal treatment option for OKCs remains controversial [9]. Because the cyst wall is thin and fragile and OKCs often contain satellite cysts, complete removal via simple enucleation may be challenging [15]. Nevertheless, enucleation is the most common treatment method [21]. Blanas et al. reported recurrence rates between 17–56% after simple enucleation [22]. Resection may eliminate recurrence but carries high morbidity [22]. Wushou et al. suggested marsupialization as the initial choice for large cysts [23]. Dong et al. found that marsupialization prevented inferior alveolar nerve paresthesia in large cysts [24]. Park et al. recommended continuing marsupialization until the cyst shrinks enough to allow safe enucleation [25]. Zhao et al. and Kubota et al. reported that large lesions shrink faster after marsupialization [26,27]. Chirapathomsakul et al. reported 22.6% recurrence, while Myoung et al. reported 58.3% [15,28]. In the present study, significant differences in recurrence rates were observed among treatment modalities in the unadjusted analysis. Enucleation demonstrated the highest recurrence rate (19.2%), whereas marsupialization was associated with a lower recurrence rate (5%), and no recurrence was observed following resection. However, these findings should be interpreted with caution. Treatment allocation was not randomized and was largely based on lesion characteristics and clinical judgment. Larger lesions and lesions located in close proximity to vital anatomical structures were more likely to be managed with marsupialization, whereas extensive or aggressive lesions were more frequently treated by resection. Therefore, the observed differences in recurrence rates may partly reflect treatment-selection bias rather than the isolated effect of the treatment modality itself.

To address this limitation, multivariable Firth logistic regression and Cox proportional hazards analyses were performed. After adjustment for potential confounding variables, treatment modality was not independently associated with recurrence in the logistic regression model. Nevertheless, in the multivariable Cox model, marsupialization remained significantly associated with a reduced risk of recurrence compared with enucleation. These findings suggest a possible benefit of marsupialization in selected cases; however, causality cannot be inferred due to the retrospective nature of the study.

Long-term follow-up remains essential in the management of odontogenic keratocysts because recurrence may occur several years after treatment. In the present study, Kaplan–Meier survival analysis demonstrated a mean recurrence-free survival time of 73.31 months and a median recurrence-free survival time of 74 months. The close agreement between mean and median values suggests a relatively balanced distribution of recurrence-free follow-up times within the study cohort. Furthermore, Cox proportional hazards analysis identified follow-up duration as an independent factor associated with recurrence risk. These findings support previous recommendations advocating prolonged clinical and radiological surveillance following treatment of OKCs. Jung et al. found that 94.8% of recurrences occurred within 10 years; thus, at least 10 years of follow-up is recommended [16]. Adjunctive therapies such as 5-FU, Carnoy’s solution, and modified Carnoy’s solution may reduce recurrence [29]. Li et al. also reported ethanol-based therapy as effective [10].

Recent evidence from systematic reviews and meta-analyses supports the complexity of interpreting recurrence outcomes in odontogenic keratocysts. Gonçalves et al., in an overview of 19 systematic reviews, reported a mean recurrence rate of 16.2% and highlighted considerable variability among treatment modalities, with the highest recurrence rates generally observed following simple enucleation [30]. The authors also emphasized the overall low methodological quality of the available evidence and the lack of definitive consensus regarding the optimal treatment approach. Similarly, da Silva et al. reported that marsupialization followed by delayed enucleation may be associated with lower recurrence rates compared with enucleation alone; however, the available evidence was insufficient to establish definitive superiority of one treatment modality over another [31]. The findings of the present study are generally consistent with these observations, as marsupialization was associated with lower recurrence rates and improved recurrence-free survival outcomes. Nevertheless, treatment-related differences should be interpreted cautiously, as lesion characteristics, treatment selection, adjunctive therapies, and follow-up duration may substantially influence recurrence outcomes.

This study has several limitations that should be considered when interpreting the findings. First, the retrospective single-center design may limit the generalizability of the results and introduce the potential for selection bias. Although multivariable logistic and Cox regression analyses were performed to adjust for potential confounding factors, treatment allocation was not randomized and was primarily based on clinical characteristics such as lesion size, anatomical location, proximity to vital structures, and surgeon preference. Therefore, residual confounding cannot be completely excluded.

Second, surveillance intensity and follow-up compliance may have varied among patients. As is common in retrospective studies with long observation periods, some patients attended follow-up visits at irregular intervals, which may have influenced the timing of recurrence detection and introduced ascertainment bias despite the use of time-to-event analyses.

Third, complete information regarding adjunctive treatment procedures, including peripheral ostectomy, Carnoy’s solution, modified Carnoy’s solution, 5-fluorouracil application, and other supplementary interventions, was not consistently available in the archival records. Because these procedures may influence recurrence outcomes, their potential effect could not be fully evaluated.

Finally, several potentially relevant radiographic and clinical variables, such as cortical perforation, soft tissue extension, multilocularity, and other lesion-specific characteristics, were not consistently documented throughout the study period and therefore could not be incorporated into the analyses. Future multicenter prospective studies with standardized treatment protocols and follow-up schedules are needed to further clarify the factors influencing recurrence and treatment outcomes in odontogenic keratocysts.

## 5. Conclusions

The management of odontogenic keratocysts remains challenging because recurrence is influenced by multiple factors, including patient characteristics, lesion-related factors, and treatment modality. In the present study, marsupialization was associated with lower recurrence rates and improved recurrence-free survival compared with enucleation. However, because of the retrospective design and non-randomized treatment allocation, these findings should be interpreted with caution and should not be considered evidence of a causal relationship.

Careful treatment planning and long-term follow-up remain essential for the management of odontogenic keratocysts, given their potential for delayed recurrence. Further prospective studies with standardized treatment protocols are required to better clarify the impact of treatment modality on recurrence outcomes.

## Figures and Tables

**Table 1:** Demographic and Clinical Characteristics According to Treatment Groups.

		Treatment Groups											
		Enucleation			Marsupialization			Resection			Total		
		n	%R	%C	n	%R	%C	n	%R	%C	n	%R	%C
Gender	Female	78	76.5	33.3	15	14.7	37.5	9	8.8	52.9	102	100.0	35.1
	Male	156	82.5	66.7	25	13.2	62.5	8	4.2	47.1	189	100.0	64.9
Age	0–10	1	100.0	0.4	0	0.0	0.0	0	0.0	0.0	1	100.0	0.3
	11–20	15	71.4	6.4	5	23.8	12.5	1	4.8	5.9	21	100.0	7.2
	21–30	39	76.5	16.7	7	13.7	17.5	5	9.8	29.4	51	100.0	17.5
	31–40	50	78.1	21.4	9	14.1	22.5	5	7.8	29.4	64	100.0	22.0
	41–50	55	83.3	23.5	10	15.2	25.0	1	1.5	5.9	66	100.0	22.7
	51–60	40	81.6	17.1	7	14.3	17.5	2	4.1	11.8	49	100.0	16.8
	61–70	29	87.9	12.4	2	6.1	5.0	2	6.1	11.8	33	100.0	11.3
	71–80	5	83.3	2.1	0	0.0	0.0	1	16.7	5.9	6	100.0	2.1
Localization	Right maxillary posterior	10	62.5	4.3	6	37.5	15.0	0	0.0	0.0	16	100.0	5.5
	Maxillary anterior	21	87.5	9.0	3	12.5	7.5	0	0.0	0.0	24	100.0	8.2
	Left maxillary posterior	11	91.7	4.7	1	8.3	2.5	0	0.0	0.0	12	100.0	4.1
	Left mandibular posterior	71	79.8	30.3	15	16.9	37.5	3	3.4	17.6	89	100.0	30.6
	Mandibular anterior	23	82.1	9.8	1	3.6	2.5	4	14.3	23.5	28	100.0	9.6
	Right mandibular posterior	94	80.3	40.2	14	12.0	35.0	9	7.7	52.9	117	100.0	40.2
	Maxillary sinus	4	80.0	1.7	0	0.0	0.0	1	20.0	5.9	5	100.0	1.7
Pre-diagnosis	Others	95	88.8	40.6	11	10.3	27.5	1	0.9	5.9	107	100.0	36.8
	Odontogenic Keratocyst	139	75.5	59.4	29	15.8	72.5	16	8.7	94.1	184	100.0	63.2
Pre-diagnosis (detail)	Odontogenic Keratocyst	139	75.5	59.4	29	15.8	72.5	16	8.7	94.1	184	100.0	63.2
	Ameloblastoma	1	100.0	0.4	0	0.0	0.0	0	0.0	0.0	1	100.0	0.3
	Dentigerous cyst	43	86.0	18.4	6	12.0	15.0	1	2.0	5.9	50	100.0	17.2
	Odontogenic cyst	14	93.3	6.0	1	6.7	2.5	0	0.0	0.0	15	100.0	5.2
	Radicular cyst	35	89.7	15.0	4	10.3	10.0	0	0.0	0.0	39	100.0	13.4
	Residual cyst	1	100.0	0.4	0	0.0	0.0	0	0.0	0.0	1	100.0	0.3
	Infected sinus mucosa	1	100.0	0.4	0	0.0	0.0	0	0.0	0.0	1	100.0	0.3
Treatment	Enucleation	234	100.0	100.0	0	0.0	0.0	0	0.0	0.0	234	100.0	80.4
	Marsupialization	0	0.0	0.0	40	100.0	100.0	0	0.0	0.0	40	100.0	13.7
	Resection	0	0.0	0.0	0	0.0	0.0	17	100.0	100.0	17	100.0	5.8
Recurrence	No recurrence	189	77.5	80.8	38	15.6	95.0	17	7.0	100.0	244	100.0	83.8
	Recurrence	45	95.7	19.2	2	4.3	5.0	0	0.0	0.0	47	100.0	16.2
Subtype	Parakeratinized	217	80.4	92.7	40	14.8	100.0	13	4.8	76.5	270	100.0	92.8
	Orthokeratinized	17	81.0	7.3	0	0.0	0.0	4	19.0	23.5	21	100.0	7.2
	Total	234	80.4	100.0	40	13.7	100.0	17	5.8	100.0	291	100.0	100.0

**Table 2:** Association between age groups and recurrence.

Age	Recurrence									Test Statistics	*p*
	-			+			Total				
	n	Row%	Column%	n	Row%	Column%	n	Row%	Column%		
0–10	0	0.0	0.0	1	100.0	2.1	1	100.0	0.3	9.235	0.215
11–20	16	76.2	6.6	5	23.8	10.6	21	100.0	7.2		
21–30	40	78.4	16.4	11	21.6	23.4	51	100.0	17.5		
31–40	55	85.9	22.5	9	14.1	19.1	64	100.0	22.0		
41–50	55	83.3	22.5	11	16.7	23.4	66	100.0	22.7		
51–60	41	83.7	16.8	8	16.3	17.0	49	100.0	16.8		
61–70	31	93.9	12.7	2	6.1	4.3	33	100.0	11.3		
71–80	6	100.0	2.5	0	0.0	0.0	6	100.0	2.1		
Total	244	83.8	100.0	47	16.2	100.0	291	100.0	100.0		

Row%: Row percentage, Column%: Column percentage.

**Table 3:** Association between lesion localization and recurrence.

Localization	Recurrence									Test Statistics	*p*
	-			+			Total				
	n	Row%	Column%	n	Row%	Column%	n	Row%	Column%		
Right maxillary posterior	14	87.5	5.7	2	12.5	4.3	16	100.0	5.5	3.751	0.698
Maxillary anterior	19	79.2	7.8	5	20.8	10.6	24	100.0	8.2		
Left maxillary posterior	12	100.0	4.9	0	0.0	0.0	12	100.0	4.1		
Left mandibular posterior	74	83.1	30.3	15	16.9	31.9	89	100.0	30.6		
Mandibular anterior	25	89.3	10.2	3	10.7	6.4	28	100.0	9.6		
Right mandibular posterior	96	82.1	39.3	21	17.9	44.7	117	100.0	40.2		
Maxillary sinus	4	80.0	1.6	1	20.0	2.1	5	100.0	1.7		
Total	244	83.8	100.0	47	16.2	100.0	291	100.0	100.0		

Row%: Row percentage, Column%: Column percentage.

**Table 4:** Association Between Histopathological Subtype and Recurrence.

Subtype	Recurrence									Test Statistics	*p*
	-			+			Total				
	n	Row%	Column%	n	Row%	Column%	n	Row%	Column%		
Parakeratinized	228	84.4	93.4	42	15.6	89.4	270	100.0	92.8	-	0.353
Orthokeratinized	16	76.2	6.6	5	23.8	10.6	21	100.0	7.2		
Total	244	83.8	100.0	47	16.2	100.0	291	100.0	100.0		

Row%: Row percentage, Column%: Column percentage.

**Table 5:** Association Between Pre-Diagnosis and Recurrence.

Pre-Diagnosis	Recurrence									Test Statistics	*p*
	-			+			Total				
	n	Row%	Column%	n	Row%	Column%	n	Row%	Column%		
Odontogenic Keratocyst	153	83.2	62.7	31	16.8	66.0	184	100.0	63.2	7.031	0.328
Ameloblastoma	1	100.0	0.4	0	0.0	0.0	1	100.0	0.3		
Dentigerous cyst	40	80.0	16.4	10	20.0	21.3	50	100.0	17.2		
Odontogenic cyst	14	93.3	5.7	1	6.7	2.1	15	100.0	5.2		
Radicular cyst	35	89.7	14.3	4	10.3	8.5	39	100.0	13.4		
Residual cyst	0	0.0	0.0	1	100.0	2.1	1	100.0	0.3		
Infected sinus mucosa	1	100.0	0.4	0	0.0	0.0	1	100.0	0.3		

Row%: Row percentage, Column%: Column percentage.

**Table 6:** Association between treatment modality and recurrence.

Treatment	Recurrence									Test Statistics	*p*
	-			+			Total				
	n	Row%	Column%	n	Row%	Column%	n	Row%	Column%		
Enucleation	189 ^a^	80.8	77.5	45 ^b^	19.2	95.7	234	100.0	80.4	X^2^ = 8.586	0.014 *
Marsupialization	38 ^a^	95.0	15.6	2 ^b^	5.0	4.3	40	100.0	13.7		
Resection	17 ^a^	100.0	7.0	0 ^a^	0.0	0.0	17	100.0	5.8		
Total	244	83.8	100.0	47	16.2	100.0	291	100.0	100.0		

* *p* < 0.05, Row%: Row percentage, Column%: Column percentage. Different superscript letters (a, b) indicate statistically significant differences between treatment groups.

**Table 7:** Distribution of Follow-Up Duration, Time to Recurrence, and Lesion Dimensions According to Treatment Groups.

	Treatment		
	Enucleation	Marsupialization	Resection
	Mean ± SD. (M., IQR)	Mean ± SD. (M., IQR)	Mean ± SD. (M., IQR)
Follow-up duration (months)	56.49 ± 13.18 (59, 12)	59.3 ± 9.02 (61, 14)	71.18 ± 5.89 (72, 8)
Time to recurrence (months)	37.4 ± 7.8 (38, 9)	36 ± 2.83 (36, 4)	0 ± 0 (0, 0)
Lesion width (mm)	14.04 ± 1.96 (14, 2)	28.65 ± 4.2 (28, 5.5)	43.94 ± 5.37 (44, 6)
Lesion length (mm)	20.38 ± 2.21 (20, 3)	41.2 ± 5.16 (41, 6.5)	62.18 ± 6.92 (62, 7)

**Table 8:** Univariate and Multivariable Firth Logistic Regression Analysis of Factors Associated with Recurrence.

Variables	Univariate OR (%95 CI)	*p*	Multivariable OR (%95 CI)	*p*
Gender (r: female)				
Male	1.182 (0.616–2.357)	0.623	0.974 (0.455–2.138)	0.947
Age (r: ≤20 years)				
21–30	0.539 (0.213–1.287)	0.173	0.5 (0.175–1.359)	0.176
31–40	0.659 (0.276–1.518)	0.333	0.763 (0.277–2.07)	0.595
41–50	0.643 (0.242–1.593)	0.353	0.524 (0.177–1.469)	0.221
51–60	0.213 (0.032–0.808)	0.047 *	0.292 (0.051–1.174)	0.085
≥61 years	0 (NA)	0.987	0.273 (0.002–3.077)	0.336
Localization (r: Maxillary anterior)				
Mandibular anterior	0.456 (0.085–2.092)	0.321	0.697 (0.114–3.917)	0.682
Right maxillary posterior	0.543 (0.07–2.936)	0.501	0.729 (0.079–5.185)	0.759
Right mandibular posterior	0.831 (0.294–2.728)	0.740	1.156 (0.357–4.28)	0.815
Maxillary sinus	0.95 (0.043–8.492)	0.967	1.098 (0.045–19.447)	0.951
Left maxillary posterior	0 (0–323,081,742,092,232)	0.989	0.135 (0.001–1.905)	0.154
Left mandibular posterior	0.77 (0.26–2.603)	0.651	0.849 (0.25–3.224)	0.800
Pre-diagnosis (r: others)				
Odontogenic keratocyst	1.152 (0.605–2.267)	0.672	1.233 (0.589–2.651)	0.581
Subtype (r: parakeratinized)				
Orthokeratinized	0.589 (0.217–1.879)	0.327	0.859 (0.218–3.666)	0.831
Treatment (r: enucleation)				
Marsupialization	0.221 (0.035–0.76)	0.043	0.092 (0.002–2.425)	0.157
Resection	0 (NA)	0.987	0.005 (0–4.857)	0.138
Follow-up duration	1.109 (1.066–1.16)	<0.001 *	1.132 (1.079–1.196)	<0.001 *
Lesion width	0.905 (0.828–0.964)	0.009 *	1.004 (0.789–1.284)	0.973
Lesion length	0.925 (0.861–0.972)	0.01 *	1.032 (0.829–1.279)	0.772

Note: OR, odds ratio; CI, confidence interval; r, reference category; NA, not available because no recurrence event occurred in the corresponding category. * *p* < 0.05.

**Table 9:** Recurrence-Free Survival Estimates.

Survival Measure	Estimate (Months)	Standard Error	95% CI Lower Limit	95% CI Upper Limit
Mean Recurrence-Free Survival Time	73.31	0.83	71.67	74.94
Median Recurrence-Free Survival Time	74	1.95	70.18	77.82

Abbreviations: CI, Confidence Interval.

**Table 10:** Assessment of the Proportional Hazards Assumption Using Schoenfeld Residuals.

Variable	χ^2^	df	*p*
Gender	0	1	0.99
Age	3.73	5	0.59
Localization	3.25	6	0.78
Pre-diagnosis	0.133	1	0.72
Subtype	2.17	1	0.14
Treatment	3.04	2	0.22
Lesion width	1.95	1	0.16
Lesion length	2.34	1	0.13
Global	11.3	18	0.88

**Table 11:** Univariate and Multivariable Cox Proportional Hazards Regression Analysis for Recurrence-Free Survival.

Variables	Univariate HR (%95 CI)	*p*	Multivariable HR (%95 CI)	*p*
Gender (r: female)				
Male	1.114 (0.601–2.066)	0.732	0.884 (0.432–1.811)	0.737
Age (r: ≤20 years)				
21–30	0.681 (0.302–1.533)	0.353	0.75 (0.293–1.92)	0.549
31–40	2.358 (1.024–5.431)	0.044	1.88 (0.731–4.835)	0.19
41–50	1.108 (0.466–2.634)	0.817	0.963 (0.384–2.414)	0.936
51–60	0.676 (0.153–2.99)	0.606	0.699 (0.151–3.239)	0.647
≥61 years	0 (0–NA)	0.996	0 (0–NA)	0.999
Localization (r: Maxillary anterior)				
Mandibular anterior	0.678 (0.162–2.842)	0.595	1.081 (0.218–5.367)	0.924
Right maxillary posterior	0.752 (0.145–3.904)	0.735	1.9 (0.24–15.063)	0.544
Right mandibular posterior	1.08 (0.406–2.868)	0.878	2.281 (0.691–7.526)	0.176
Maxillary sinus	1.175 (0.137–10.108)	0.883	1.48 (0.124–17.672)	0.757
Left maxillary posterior	0 (0–NA)	0.996	0 (0–NA)	0.999
Left mandibular posterior	0.978 (0.354–2.698)	0.965	1.432 (0.438–4.687)	0.553
Pre-diagnosis (r: others)				
Odontogenic keratocyst	0.779 (0.424–1.431)	0.421	1.054 (0.527–2.107)	0.882
Subtype (r: parakeratinized)				
Orthokeratinized	1.176 (0.459–3.013)	0.735	1.304 (0.38–4.478)	0.673
Treatment (r: enucleation)				
Marsupialization	0.242 (0.058–1)	0.05	0.02 (0.001–0.68)	0.03
Resection	0 (NA)	0.997	0 (NA)	0.995
Follow-up duration	1.109 (1.066–1.16)	<0.001 *	1.132 (1.079–1.196)	<0.001 *
Lesion width	0.906 (0.856–0.958)	<0.001 *	0.901 (0.718–1.132)	0.371
Lesion length	0.93 (0.891–0.97)	<0.001 *	1.204 (0.977–1.483)	0.081

Note: HR, hazard ratio; CI, confidence interval; r, reference category; NA, not available because no recurrence event occurred in the corresponding category. * *p* < 0.05.

## Data Availability

Data are contained within the article.

## Abbreviations

The following abbreviations are used in this manuscript:

OKC	Odontogenic Keratocyst
WHO	World Health Organization
NBCCS	Nevoid Basal Cell Carcinoma Syndrome
5-FU	5-Fluorouracil
SPSS	Statistical Package for the Social Sciences
